# Periodontal therapy for localized severe periodontitis in a patient receiving fixed orthodontic treatment: a case report

**DOI:** 10.1186/s13256-023-03751-1

**Published:** 2023-01-20

**Authors:** Satoru Morikawa, Kazuya Watanabe, Ryo Otsuka, Seiji Asoda, Taneaki Nakagawa

**Affiliations:** 1 Department of Dentistry and Oral Surgery, Keio University School of Medicine, 35 Shinanomachi, Shinjuku-ku, Tokyo, 160-8582 Japan; 2 Watanabe Orthodontic Office, 1-11-26-2F Kichijoji-honcho, Musashino, Tokyo 180-0004 Japan; 3 Familia Orthodontics, 1-7-5-12F Sakuragi-cho, Omiya-ku, Saitama, Saitama 330-0854 Japan

**Keywords:** Orthodontic treatment, Periodontal examination, Severe periodontitis, Case report

## Abstract

**Background:**

Orthodontic treatment involves movement of teeth by compression and resorption of the alveolar bone using orthodontic forces. These movements are closely linked to the interactions between the teeth and the periodontal tissues that support them. Owing to an increase in adults seeking orthodontic treatment, orthodontists increasingly encounter patients with periodontal diseases, in whom orthodontic treatment is contraindicated. In rare cases, periodontitis may develop after treatment initiation. However, no approach for treating periodontitis after the initiation of orthodontic treatment has been established. Here, we present an approach for managing localized severe periodontitis manifesting after initiating orthodontic treatment.

**Case presentation:**

A 32-year-old Japanese woman was referred to the Department of Dentistry and Oral Surgery by an orthodontist who observed symptoms of acute periodontitis in the maxillary molars that required periodontal examination and treatment. A detailed periodontal examination, including oral bacteriological examination, revealed localized severe periodontitis (stage III, grade B) in the maxillary left first and second molars and in the mandibular right second molar. After consultation with the orthodontist, the orthodontic treatment was suspended based on the results of the bacteriological examination to allow for periodontal treatment. Full-mouth disinfection was performed with adjunctive oral sitafloxacin. Periodontal and bacteriological examinations after treatment revealed regression of the localized periodontitis with bone regeneration. Thereafter, orthodontic treatment was resumed, and good progress was achieved.

**Conclusions:**

Orthodontists should recognize the risk of acute severe periodontitis in young adults. Asymptomatic patients with localized severe periodontitis may clear a screening test before orthodontic treatment but develop acute symptoms with bone resorption during orthodontic treatment. Therefore, patients requiring orthodontic treatment should be examined by their family dentist or a periodontist to rule out periodontal issues that may impede orthodontic treatment. The patients should also be informed of age-related risks. Further, periodontists, family dentists, and orthodontists who treat adults should be informed about periodontitis and the need for interdisciplinary collaboration. In patients who develop periodontitis after orthodontic treatment initiation, temporary interruption of orthodontic treatment and aggressive periodontal intervention may facilitate recovery.

## Background

The objective of orthodontic treatment is to provide a functional and cosmetic dental occlusion through optimal movements of the teeth. These movements are closely linked to interactions between the teeth and the periodontal tissues that support them. As the number of adult patients seeking orthodontic treatment has increased in recent years, orthodontists are increasingly encountering individuals with periodontal disease. If periodontal inflammation is not controlled during orthodontic treatment, periodontal damage can progress rapidly, leading to additional attachment loss [1]. However, an approach for treating periodontitis after the initiation of orthodontic treatment has not yet been established.

The concept of single-stage full-mouth disinfection (FMD), proposed > 25 years ago [2], has been advocated for its therapeutic and microbiological benefits [3, 4]. It is based on the discovery that periodontal bacteria from untreated periodontal pockets and oral niches rapidly recolonize newly treated periodontal pockets [5].

To achieve optimal treatment outcomes in modern clinical practice, interdisciplinary treatment should be performed by orthodontists, periodontists, and general dentists. We report a case of successful collaboration between a periodontist and orthodontist to treat localized stage III periodontitis that manifested after initiation of orthodontic treatment by applying FMD combined with systemic antimicrobial therapy.

## Case presentation

A 32-year-old Japanese woman was referred to our department due to complaints of spontaneous gingival pain, bleeding, and increased tooth mobility of the maxillary left second molar. The patient had already started orthodontic treatment at the time of referral; she had acute seizure symptoms during orthodontic treatment and was receiving antimicrobial medications at the time of presentation. According to the referring orthodontist, the patient had been screened by her family dentist (general practitioner) for caries and periodontal disease before orthodontic treatment, and orthodontic treatment was initiated based on the results of the screening. The patient had been experiencing gingival inflammation since the initiation of the orthodontic treatment, and emergency treatment had been provided in collaboration with her family dentist. However, the patient’s symptoms of pain, gingival swelling, and tooth mobility worsened, and her family dentist suggested the need for specialized diagnosis and treatment by the orthodontist, who in turn referred the patient.

### Investigations

**Fig. 1:**
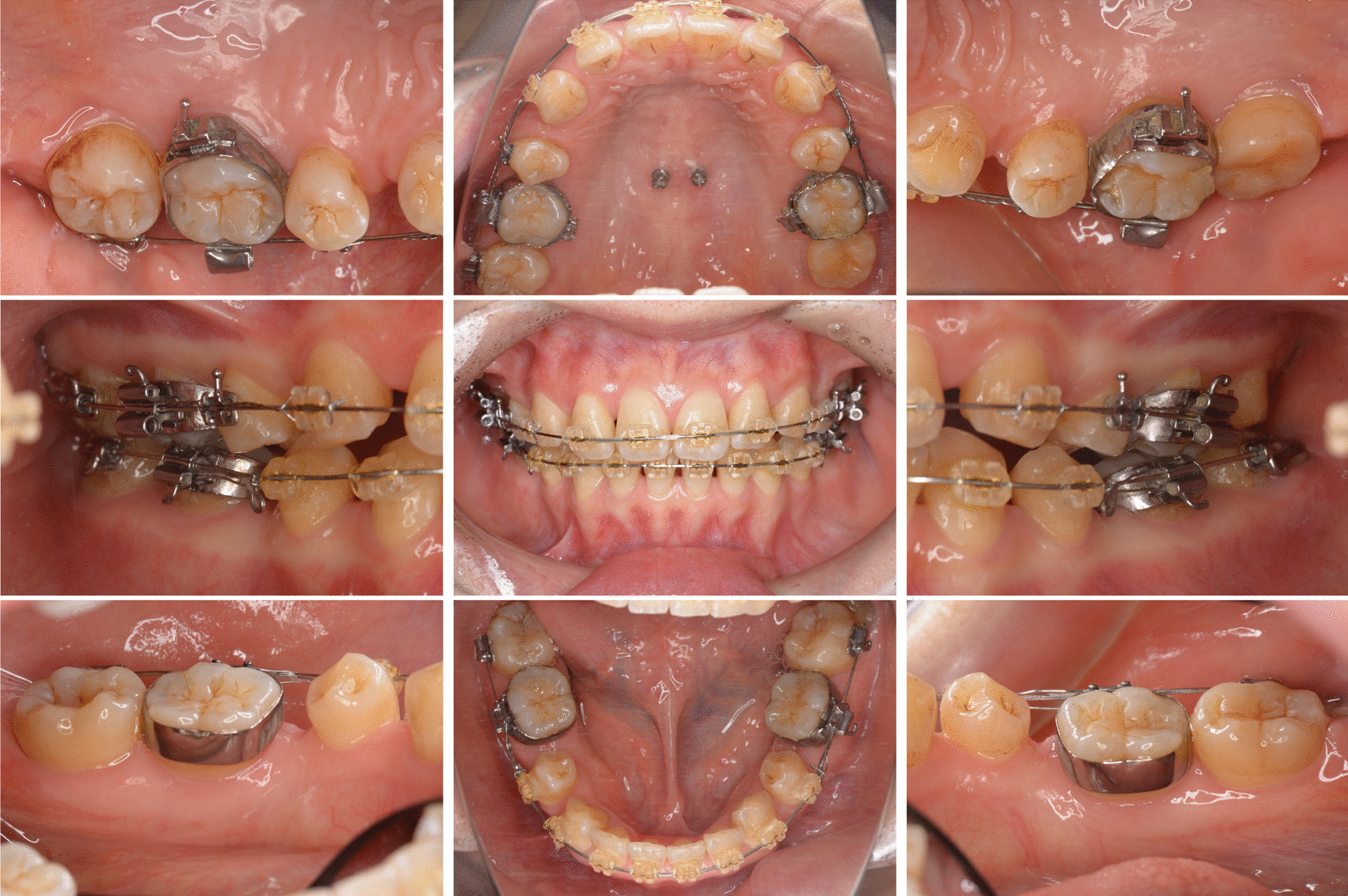
Clinical appearance before treatment. No significant gingival redness or swelling can be observed

**Fig. 2:**
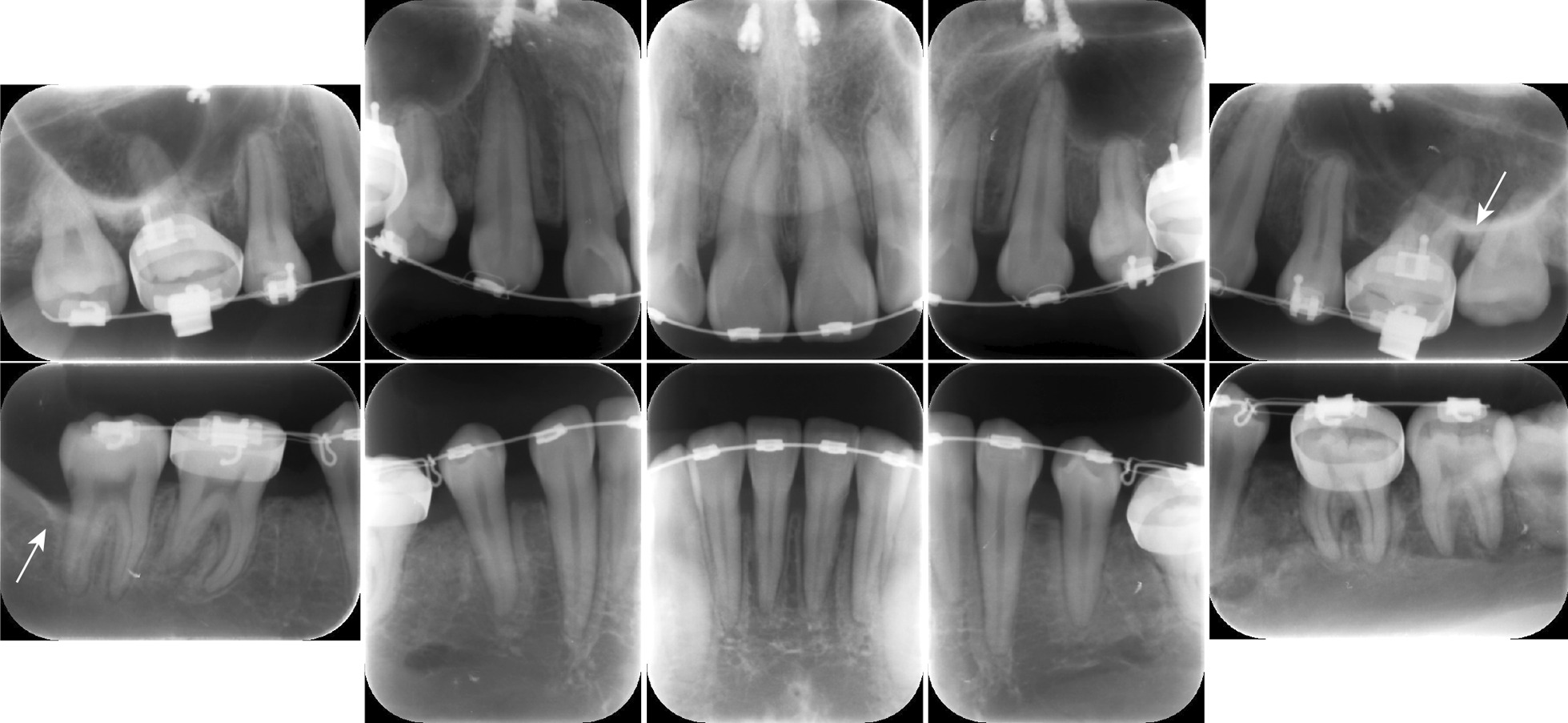
Intraoral periapical radiographs. White arrows indicate vertical bone loss

To make a diagnosis and develop a treatment plan, we performed oral and periodontal examinations. Periodontal damage was diagnosed based on probing pocket depth (PPD) measurements at six sites around each tooth and evaluations of the teeth and soft tissues (Fig. 1). Intraoral periapical radiographs revealed extensive alveolar bone loss (Fig. 2), including vertical bone resorption extending from the distal aspect of the maxillary left first molar to the mesial aspect of the maxillary left second molar. Vertical bone loss was also observed distal to the mandibular right second molar (Fig. 2, white arrow). Clinically, deep periodontal pockets (PPD, 4–7 mm) were detected around the bilateral maxillary first and second molars and bilateral mandibular second molars. The inflamed periodontium had a surface area of 277.4 mm^2^ [6, 7]. Based on the initial periodontal findings, severe localized stage III grade B periodontitis in the maxillary left first and second molars and in the mandibular right second molar was diagnosed [7]. As the patient had good general health and only localized clinical attachment loss, we suspected localized aggressive periodontitis (1999 International Workshop for a Classification of Periodontal Disease and Conditions) in relation to the maxillary left first and second molars and the mandibular right second molar [8].

We conducted bacteriological examinations to observe the bacterial flora before and after treatment. Bacterial specimens were collected using paper points from periodontal pockets around the maxillary left second molar, the site of acute periodontal disease, and the distal side of the mandibular right second molar with vertical bone loss on dental radiographs. Saliva specimens were also collected, and real-time polymerase chain reaction analysis (Perio-Analysis; NOVEO, Hong Kong, China) was conducted (Table 1). The pretreatment bacteriological examination showed that the counts of *Porphyromonas gingivalis*, *Tannerella forsythia*, and *Treponema denticola* (red complex) as well as those of *Prevotella intermedia*, *Parvimonas micra*, and *Fusobacterium nucleatum* (orange complex) at the maxillary left second molar were > tenfold higher than the tolerated values. At the mandibular right second molar, the counts of *T. forsythia*, *T. denticola*, *P. intermedia*, *P. micra*, and *Campylobacter rectus* were > tenfold higher than the tolerable values. Similarly, in the saliva samples, the counts of *T. forsythia*, *T. denticola*, *P. micra*, *F. nucleatum*, *C. rectus*, and *Eikenella corrodens* were ten times higher than the permissible values (Table 1).

**Table 1:** Real-time polymerase chain reaction evaluation of periodontal pathogenic load at the first visit

Bacteria	Pathogenic load	Pathogenic threshold	Status
**Maxillary left second molar**			
*Porphyromonas gingivalis*	0.0E+00	1.0E+05	−
*Tannerella forsythia*	3.2E+03	1.0E+05	+
*Treponema denticola*	3.3E+05	1.0E+05	++
*Prevotella intermedia*	1.1E+06	1.0E+05	+++
*Parvimonas micra*	9.2E+03	1.0E+06	+
*Fusobacterium nucleatum*	7.6E+05	1.0E+07	+
*Campylobacter rectus*	4.1E+05	1.0E+06	+
*Eikenella corrodens*	2.4E+06	1.0E+07	+
**Mandibular right second molar**			
*Porphyromonas gingivalis*	0.0E+00	1.0E+05	−
*Tannerella forsythia*	5.3E+06	1.0E+05	+++
*Treponema denticola*	4.5E+06	1.0E+05	+++
*Prevotella intermedia*	9.3E+06	1.0E+05	+++
*Parvimonas micra*	7.9E+06	1.0E+06	++
*Fusobacterium nucleatum*	1.0E+06	1.0E+07	+
*Campylobacter rectus*	1.2E+07	1.0E+06	+++
*Eikenella corrodens*	8.2E+06	1.0E+07	+
**Saliva**			
*Porphyromonas gingivalis*	0.0E+00	1.0E+05	−
*Tannerella forsythia*	3.8E+06	1.0E+05	+++
*Treponema denticola*	6.1E+06	1.0E+05	+++
*Prevotella intermedia*	0.0E+00	1.0E+05	-
*Parvimonas micra*	1.3E+08	1.0E+06	+++
*Fusobacterium nucleatum*	5.7E+08	1.0E+07	+++
*Campylobacter rectus*	3.4E+08	1.0E+06	+++
*Eikenella corrodens*	1.2E+09	1.0E+07	+++

The counts of several bacteria are > ten times the pathogenic load threshold. The pathogenic load is the quantity of detected bacteria in a sample. The pathogenic threshold represents a specific microbiological pathogenic load above which antibiotic therapy is recommended to reduce the risk of tooth or implant attachment loss (periodontal disease or peri-implantitis). Status refers to the levels of microbiological pathogenic load: − absent; + moderate and less than the pathogenic load threshold; ++ high and more than the pathogenic load threshold and associated with aggressive forms of disease; +++ very high (> ten times the pathogenic load threshold) and strongly associated with aggressive forms of disease and loss of bone attachment

### Treatment

To eliminate the etiology of the periodontal disease, the orthodontist treating the patient was consulted and all orthodontic treatments were suspended. The orthodontic appliance was removed from the upper and lower molars in the affected area. FMD was performed within 24 hours of debridement of all periodontal pockets by scaling and root planing (SRP) using Gracey curettes, followed by adjunctive oral sitafloxacin (100 mg/day for 7 days). The patient was educated on the Bass technique for toothbrushing and interdental brushing techniques, and detailed oral hygiene instructions were given to ensure a significant reduction in bacterial load. Plaque was also found around the brackets; therefore, we educated the patient to identify sites of plaque accumulation and instructed her on how to carefully remove it.

### Outcome and follow-up

**Fig. 3:**
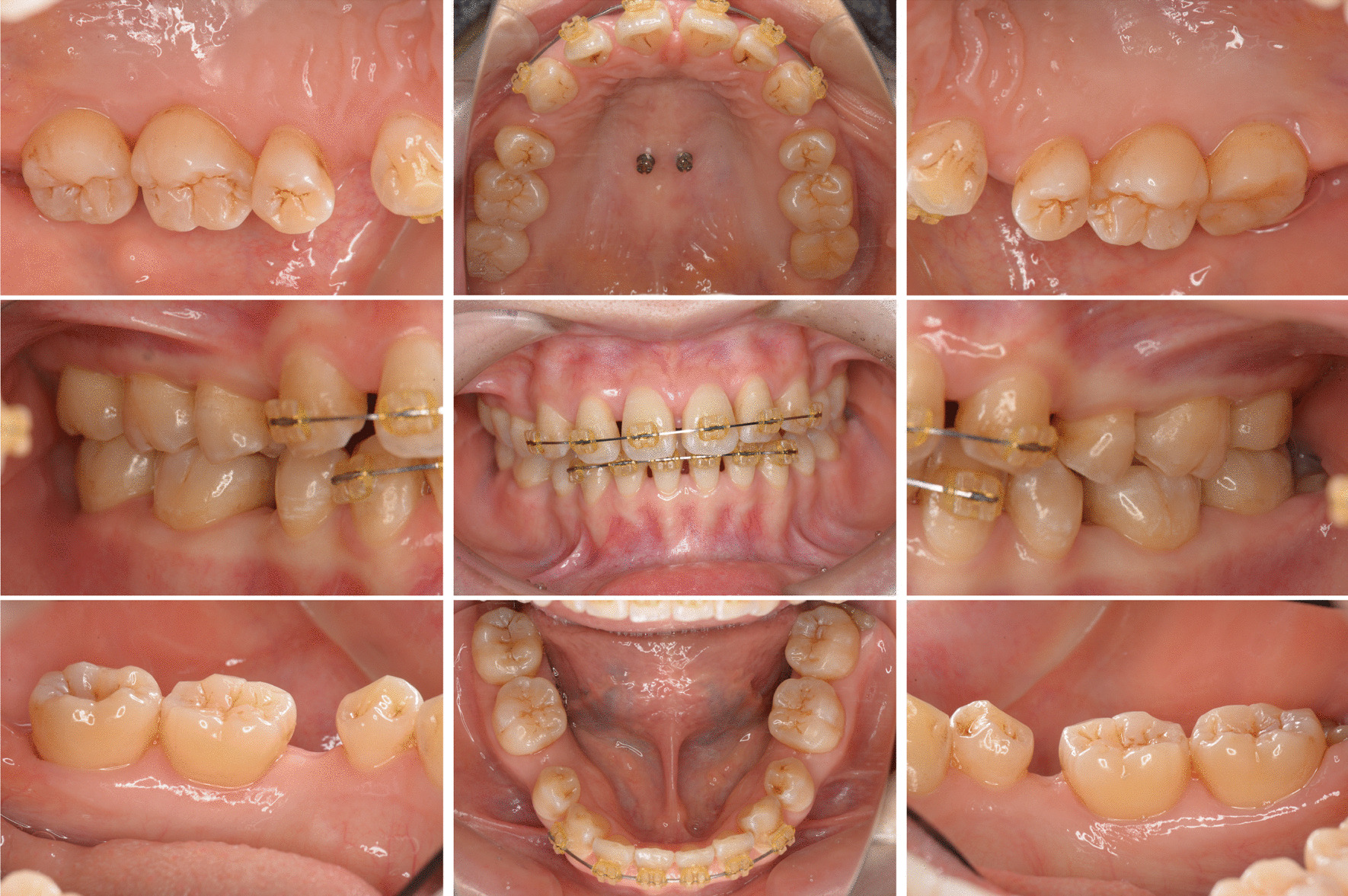
Intraoral clinical images after periodontal therapy. Signs of inflammation are absent

**Fig. 4:**
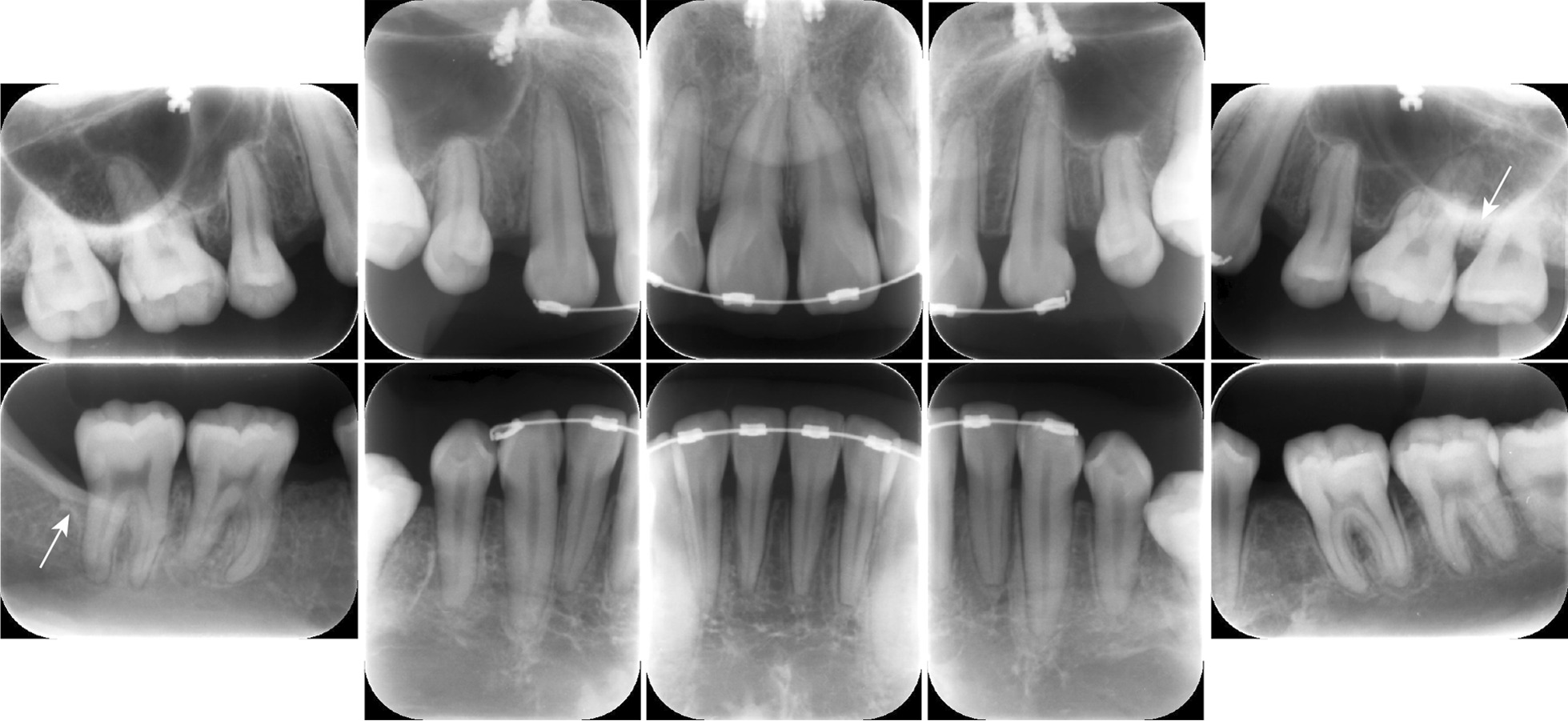
Intraoral periapical radiographs after periodontal treatment. No evidence of bone resorption progression is observed. Alveolar bone deposition can be observed on the distal aspects of the maxillary left second molar and mandibular right second molar (white arrows), thus, indicating that bone grafting is not required

At 3 months after FMD, we observed no recurrence of periodontal disease (Fig. 3). Post-treatment radiographs revealed that there was no advancement in alveolar bone resorption since the initial examination or in alveolar bone deposition around the maxillary left second molar and the mandibular right second molar (Fig. 4, white arrows).

The O’Leary plaque control scores were improved from 49% to 22%, bleeding on probing decreased from 24 (16.7%) to 21 sites (14.6%), the average PPD was improved from 2.4 to 2.1 mm, and periodontal inflammatory surface area decreased from 277.4 to 125.6 mm^2^. The patient is currently receiving supportive periodontal therapy, and her condition is stable without recurrence of periodontal disease. Orthodontic treatment was put on hold for 3 months during the primary periodontal treatment phase; however, it was resumed at the same time as was the supportive periodontal therapy.

The counts of *P. intermedia* and *C. rectus* in the saliva samples were only ten times higher than the reference value, which was one of the reasons for periodontal disease management with supportive periodontal therapy. The red complex was no longer detected in the periodontal pockets that showed severe disease before treatment, and the orange-complex counts decreased below the standard value. However, in the saliva samples, *T. denticola* was detected, and the orange-complex counts, especially the *P. intermedia* count, were approximately 100 times higher than the reference values (Table 2).

**Table 2:** Real-time polymerase chain reaction evaluation of periodontal pathogenic load after periodontal treatment

Bacteria	Pathogenic load	Pathogenic threshold	Status
**Maxillary left second molar**			
*Porphyromonas gingivalis*	0.0E+00	1.0E+05	−
*Tannerella forsythia*	0.0E+00	1.0E+05	−
*Treponema denticola*	0.0E+00	1.0E+05	−
*Prevotella intermedia*	8.6E+03	1.0E+05	+
*Parvimonas micra*	2.3E+04	1.0E+06	+
*Fusobacterium nucleatum*	3.8E+04	1.0E+07	+
*Campylobacter rectus*	3.0E+04	1.0E+06	+
*Eikenella corrodens*	7.8E+03	1.0E+07	+
**Mandibular right second molar**			
*Porphyromonas gingivalis*	0.0E+00	1.0E+05	−
*Tannerella forsythia*	0.0E+00	1.0E+05	−
*Treponema denticola*	0.0E+00	1.0E+05	−
*Prevotella intermedia*	0.0E+00	1.0E+05	−
*Parvimonas micra*	2.8E+04	1.0E+06	+
*Fusobacterium nucleatum*	1.3E+06	1.0E+07	+
*Campylobacter rectus*	1.8E+05	1.0E+06	+
*Eikenella corrodens*	1.1E+05	1.0E+07	+
**Saliva**			
*Porphyromonas gingivalis*	0.0E+00	1.0E+05	−
*Tannerella forsythia*	0.0E+00	1.0E+05	−
*Treponema denticola*	2.5E+04	1.0E+05	+
*Prevotella intermedia*	8.9E+06	1.0E+05	+++
*Parvimonas micra*	1.8E+06	1.0E+06	++
*Fusobacterium nucleatum*	9.5E+07	1.0E+07	++
*Campylobacter rectus*	1.8E+07	1.0E+06	+++
*Eikenella corrodens*	1.3E+07	1.0E+07	++

The pathogenic load in the periodontal pockets is below the threshold

Currently, the patient has resumed orthodontic treatment, which is progressing well with no evidence of recurrence of periodontal disease.

## Discussion and conclusions

We encountered a case of localized periodontitis (stage III, grade B) during orthodontic treatment. The current case presents two significant considerations for clinicians. First, localized stage III periodontitis, which may be missed during initial examination, can become exacerbated during orthodontic treatment. Exacerbation may require temporary interruption of orthodontic treatment to allow resolution of active periodontitis through periodontal intervention. Second, patients who have undergone periodontal treatment should be carefully examined by a periodontist during orthodontic treatment. Orthodontists should monitor the periodontal tissue carefully during orthodontic treatment and collaborate with periodontists as and when necessary.

Pre-orthodontic oral examination is often performed by general dentists, and not all cases are evaluated by periodontists using periapical radiographs (the Dental X-P 10 or 14 card method) to thoroughly assess the bone levels. Panoramic radiography and basic periodontal examination before proceeding with orthodontic treatment are probably more common if the patient has no relevant complaints. However, as it is uncommon for a dentist to have expertise in both orthodontics and periodontics, localized periodontitis that may not require periodontal surgery, such as in this case, may be overlooked before orthodontic treatment.

Fixed orthodontic treatment has minimal to no significant effects on the clinical attachment levels (periodontal conditions) in healthy adults and adolescents [9, 10]. However, in cases of localized moderate periodontitis with no obvious symptoms, as in the present case, orthodontic treatment may induce acute symptoms and bone resorption. If periodontal inflammation is not controlled during orthodontic treatment, periodontal damage can progress rapidly, leading to additional attachment loss [1]. In the worst-case scenario, the orthodontic treatment may have to be interrupted. However, it is possible to recover from such a situation with accurate diagnosis and professional treatment.

We performed a thorough clinical and radiographic evaluation of individual teeth in addition to bacteriological examination of the sites with deep periodontal pockets. The World Workshop in Periodontology (1999) defined aggressive periodontitis as “a type of periodontitis that typically affects young people with a non-contributory medical history and familial aggregation, resulting in rapid attachment loss and bone resorption” [8]. According to the consensus report from the International Workshop on the Classification of Periodontal Diseases and Conditions, an increase in the prevalence of *Porphyromonas gingivalis* and *Aggregatibacter actinomycetemcomitans* in some populations is a potential secondary feature of localized and generalized aggressive periodontitis [11]. As some periodontal bacteria with high pathogenicity contribute to the development of aggressive periodontitis [12] and affect its prognosis, we performed bacteriological examination. *Treponema denticola* has been increasingly detected in patients with aggressive periodontitis [13], and according to the 1999 American Association of Periodontology classification of periodontitis [8], continued supportive periodontal therapy after orthodontic treatment is necessary to prevent recurrence of periodontal disease.

In the present case, we aimed to complete periodontal treatment and resume orthodontic treatment as soon as possible. We administered systemic antibiotic therapy along with FMD to effectively treat severe generalized periodontitis within a short period [14]. We selected FMD for the following reasons: (a) real-time polymerase chain reaction showed that the counts of periodontitis-related pathogenic bacteria in the maxillary left second molar area, mandibular right second molar area, and saliva were > ten times higher than the reference value; (b) to minimize the risk of reinfection from the SRP-treated sites to untreated sites; (c) to achieve timely completion of periodontal treatment and to resume orthodontic treatment as soon as possible; and (d) to prevent or minimize bacteremia and a rise in body temperature, which are possible adverse complications of FMD [15, 16]. To achieve these objectives, periodontal disease elimination therapy was performed at sites with PPD ≥ 4 mm, which were confined to the molars. In accordance with the original protocol, FMD was completed within 24 hours [2, 15, 17]. Oral sitafloxacin, which is effective against periodontitis-related pathogenic bacteria, was administered as an adjunctive therapy [18].

FMD minimizes the risk of reinfection from untreated periodontal pockets. Its clinical effects have been extensively researched [4, 19–21]; however, the synergistic effect of antibiotic or chlorhexidine treatment with FMD is debatable. The combination of chlorhexidine treatment with FMD demonstrated clinical outcomes that are superior to those of SRP per quadrant in several trials [22–24]; in other trials, the effects of FMD with or without antiseptics are comparable to those of standard SRP [25–27]. The use of 0.2% and 1% chlorhexidine is banned in Japan, preventing its use in the present case. In Japan, as cases of anaphylactic shock due to chlorhexidine gluconate have been reported, the concentration of undiluted chlorhexidine gluconate is regulated up to 0.05%. In practice, chlorhexidine gluconate is often applied at a concentration of approximately 0.01% because it is diluted for use. When bacterial factors are suspected to be more strongly associated with periodontal disease than host or environmental factors, the treatment should include FMD and adjunctive antimicrobial therapy with a limited dosage and duration of treatment. Full-mouth SRP has severe systemic effects, such as fever and a transient increase in the inflammatory cytokine levels [16]. The concomitant use of antimicrobial agents can significantly reduce bacteremia caused by SRP [28]. The combined amoxicillin and metronidazole administration is the most frequently used antimicrobial treatment during FMD [29, 30]. An earlier meta-analysis reported that the use of a combination of amoxicillin and metronidazole as an adjuvant to SRP presented therapeutic effects [31]. However, metronidazole is not indicated in Japan for infections in the oral cavity. Thus, in the present case, sitafloxacin was used, and we observed a significant post-treatment decrease in red-complex bacteria in active periodontal pockets [32, 33].

The risk of development of resistant strains due to the misuse of antimicrobial drugs must be recognized. To reduce the emergence of antibiotic-resistant strains and adverse effects, we chose a 7-day antibiotic regimen. In previous investigations on antibiotic resistance, researchers discovered that a single course of systemic antibiotics combined with mechanical debridement resulted in transitory bacterial resistance that dissipated shortly after the treatment was stopped [34, 35]. In the present study, the initial bacteriological examination had shown significant numbers of red and orange complexes. Therefore, we used sitafloxacin to eradicate these bacterial complexes and prevent bacteremia and febrile reactions, which are side effects of FMD. However, in cases where the bacterial levels are not significantly high, conventional SRP may be adequate.

Localized stage III periodontitis overlooked during screening of the patient can manifest with acute symptoms during orthodontic treatment. Our report presents two important clinical considerations in this regard. First, as orthodontic treatment often involves extraction procedures and treatment interruption is not possible in principle, orthodontists should consider a thorough periodontal diagnosis by the patient’s family dentist or a periodontist before orthodontic treatment, and general dentists should consider the possibility that their patients may be candidates for orthodontic treatment. Orthodontists should be informed on periodontitis; particularly, orthodontists treating adults must be knowledgeable regarding the management of localized severe periodontitis through interdisciplinary collaboration with periodontists. Second, if periodontitis or local acute inflammation occurs during orthodontic treatment, temporary interruption of treatment followed by diagnosis and treatment by a collaborating periodontist may facilitate recovery. In the present case, the periodontal parameters were improved, alveolar bone resorption stabilized following basic periodontal treatment with FMD and oral sitafloxacin, and the patient resumed orthodontic treatment. This case of localized periodontitis manifesting during orthodontic treatment may be rare; however, with the increase in the number of adults seeking orthodontic treatment, such cases are expected to be more common. In the future, it is desirable to establish clinical and bacteriological evaluation techniques that can be used by orthodontists, periodontists, and general dentists together.

## Data Availability

All data generated or analyzed during this study are included in the published article.

## Abbreviations

FMD: Full-mouth disinfection
PPD: Probing pocket depths
SRP: Scaling and root planning

## References

[1] Wennström JL, Stokland BL, Nyman S, Thilander B (1993). Periodontal tissue response to orthodontic movement of teeth with infrabony pockets. Am J Orthod Dentofacial Orthop.

[2] Quirynen M, Bollen CM, Vandekerckhove BN, Dekeyser C, Papaioannou W, Eyssen H (1995). Full- vs. partial-mouth disinfection in the treatment of periodontal infections: short-term clinical and microbiological observations. J Dent Res..

[3] Lang NP, Tan WC, Krähenmann MA, Zwahlen M (2008). A systematic review of the effects of full-mouth debridement with and without antiseptics in patients with chronic periodontitis. J Clin Periodontol.

[4] Fang H, Han M, Li QL, Cao CY, Xia R, Zhang ZH (2016). Comparison of full-mouth disinfection and quadrant-wise scaling in the treatment of adult chronic periodontitis: a systematic review and meta-analysis. J Periodontal Res.

[5] Petersilka GJ, Ehmke B, Flemmig TF (2000). Antimicrobial effects of mechanical debridement. Periodontol.

[6] Nesse W, Abbas F, van der Ploeg I, Spijkervet FK, Dijkstra PU, Vissink A (2008). Periodontal inflamed surface area: quantifying inflammatory burden. J Clin Periodontol.

[7] Tonetti MS, Greenwell H, Kornman KS (2018). Staging and grading of periodontitis: framework and proposal of a new classification and case definition. J Periodontol.

[8] Armitage GC (1999). Development of a classification system for periodontal diseases and conditions. Ann Periodontol.

[9] Bollen AM, Cunha-Cruz J, Bakko DW, Huang GJ, Hujoel PP (2008). The effects of orthodontic therapy on periodontal health: a systematic review of controlled evidence. J Am Dent Assoc..

[10] Papageorgiou SN, Papadelli AA, Eliades T (2018). Effect of orthodontic treatment on periodontal clinical attachment: a systematic review and meta-analysis. Eur J Orthod.

[11] Könönen E, Müller HP (2000). Microbiology of aggressive periodontitis. Periodontol.

[12] Kornman KS, Löe H (2000). The role of local factors in the etiology of periodontal diseases. Periodontol.

[13] Takeuchi Y, Umeda M, Ishizuka M, Huang Y, Ishikawa I (2003). Prevalence of periodontopathic bacteria in aggressive periodontitis patients in a Japanese population. J Periodontol.

[14] Aimetti M, Romano F, Guzzi N, Carnevale G (2012). Full-mouth disinfection and systemic antimicrobial therapy in generalized aggressive periodontitis: a randomized, placebo-controlled trial. J Clin Periodontol.

[15] Quirynen M, Mongardini C, de Soete M, Pauwels M, Coucke W, van Eldere J (2000). The role of chlorhexidine in the one-stage full-mouth disinfection treatment of patients with advanced adult periodontitis. Long-term clinical and microbiological observations. J Clin Periodontol..

[16] Morozumi T, Yashima A, Gomi K, Ujiie Y, Izumi Y, Akizuki T (2018). Increased systemic levels of inflammatory mediators following one-stage full-mouth scaling and root planing. J Periodontal Res.

[17] Quirynen M, Mongardini C, van Steenberghe D (1998). The effect of a 1-stage full-mouth disinfection on oral malodor and microbial colonization of the tongue in periodontitis. A pilot study. J Periodontol.

[18] Asoda S, Iwsaki R, Morita M, Horie N, Onizawa K, Uchiyama K (2017). Oral and maxillofacial tissue penetration of sitafloxacin following oral administration of a single 100-mg dose. Oral Sci Int.

[19] Aimetti M, Romano F, Guzzi N, Carnevale G (2011). One-stage full-mouth disinfection as a therapeutic approach for generalized aggressive periodontitis. J Periodontol.

[20] Bollen CM, Mongardini C, Papaioannou W, Van Steenberghe D, Quirynen M (1998). The effect of a one-stage full-mouth disinfection on different intra-oral niches. Clinical and microbiological observations. J Clin Periodontol..

[21] Vandekerckhove BN, Bollen CM, Dekeyser C, Darius P, Quirynen M (1996). Full- versus partial-mouth disinfection in the treatment of periodontal infections. Long-term clinical observations of a pilot study. J Periodontol..

[22] De Soete M, Mongardini C, Peuwels M, Haffajee A, Socransky S, van Steenberghe D (2001). One-stage full-mouth disinfection. Long-term microbiological results analyzed by checkerboard DNA-DNA hybridization. J Periodontol..

[23] Fonseca DC, Cortelli JR, Cortelli SC, Miranda Cota LO, Machado Costa LC, Moreira Castro MV (2015). Clinical and microbiologic evaluation of scaling and root planing per quadrant and one-stage full-mouth disinfection associated with azithromycin or chlorhexidine: a clinical randomized controlled trial. J Periodontol.

[24] Mongardini C, van Steenberghe D, Dekeyser C, Quirynen M (1999). One stage full- versus partial-mouth disinfection in the treatment of chronic adult or generalized early-onset periodontitis. I. Long-term clinical observations. J Periodontol.

[25] Apatzidou DA, Kinane DF (2004). Quadrant root planing versus same-day full-mouth root planning. I. Clinical findings. J Clin Periodontol.

[26] Apatzidou DA, Riggio MP, Kinane DF (2004). Quadrant root planing versus same-day full-mouth root planning. II. Microbiological findings. J Clin Periodontol.

[27] Eberhard J, Jepsen S, Jervøe-Storm PM, Needleman I, Worthington HV (2015). Full-mouth treatment modalities (within 24 hours) for chronic periodontitis in adults. Cochrane Database Syst Rev.

[28] Morozumi T, Kubota T, Abe D, Shimizu T, Komatsu Y, Yoshie H (2010). Effects of irrigation with an antiseptic and oral administration of azithromycin on bacteremia caused by scaling and root planing. J Periodontol.

[29] Cosgarea R, Juncar R, Heumann C, Tristiu R, Lascu L, Arweiler N (2016). Non-surgical periodontal treatment in conjunction with 3 or 7 days systemic administration of amoxicillin and metronidazole in severe chronic periodontitis patients. A placebo-controlled randomized clinical study. J Clin Periodontol..

[30] Sgolastra F, Petrucci A, Gatto R, Monaco A (2012). Effectiveness of systemic amoxicillin/metronidazole as an adjunctive therapy to full-mouth scaling and root planing in the treatment of aggressive periodontitis: a systematic review and meta-analysis. J Periodontol.

[31] Zandbergen D, Slot DE, Cobb CM, Van der Weijden FA (2013). The clinical effect of scaling and root planing and the concomitant administration of systemic amoxicillin and metronidazole: a systematic review. J Periodontol.

[32] Nakajima T, Okui T, Miyauchi S, Honda T, Shimada Y, Ito H (2012). Effects of systemic sitafloxacin on periodontal infection control in elderly patients. Gerodontology.

[33] Nakajima T, Okui T, Ito H, Nakajima M, Honda T, Shimada Y (2016). Microbiological and clinical effects of sitafloxacin and azithromycin in periodontitis patients receiving supportive periodontal therapy. Antimicrob Agents Chemother.

[34] Feres M, Haffajee AD, Allard K, Som S, Goodson JM, Socransky SS (2002). Antibiotic resistance of subgingival species during and after antibiotic therapy. J Clin Periodontol.

[35] Haffajee AD (2006). Systemic antibiotics: to use or not to use in the treatment of periodontal infections. That is the question. J Clin Periodontol..

